# 2022 Reviewer Acknowledgement

**DOI:** 10.1111/jdi.13943

**Published:** 2022-12-04

The publication of invaluable papers in the *Journal of Diabetes Investigation* depends on the prompt, careful review of submitted manuscripts. We would like to thank the Editorial Board members, the International Editorial Board members, the Assistant Editorial Board members and the following experts for reviewing manuscripts from September 1, 2021 to August 31, 2022.

Vasudha Ahuja

Jun Aida

Toru Aizawa

Satoru Akazawa

Sadanori Akita

Yutaro Akiyama

Gulali Aktas

Hitoshi Ando

Keiko Arai

Shin‐Ichi Araki

Osamu Arisaka

Shunichiro Asahara

Yoshimasa Aso

Masayuki Baba

Tetsuya Babazono

Jae Hyun Bae

J Baelde

Yuqian Bao

Neal Barshes

Ryotaro Bouchi

Xiaoling Cai

Soner Cander

Ali J. Chakera

Subrata Chakrabarti

Yi‐Cheng Chang

Harn‐Shen Chen

Yang Ching Chen

Kenneth Cheng

Ken Chiu

Nf Chu

Ohad Cohen

Qinghua Cui

Hiroyuki Daida

Makoto Daimon

Taura Daisuke

Undurti Das

Takahisa Deguchi

Kentaro Doi

Ken Ebihara

Jun Eguchi

Amany El‐Sikaily

Masanori Emoto

Joel Francis

Rumi Fujikawa

Junji Fujikura

Kei Fujimoto

Shimpei Fujimoto

Tomomi Fujisawa

Yoshihito Fujita

Yukihiro Fujita

Yoshio Fujitani

Kei Fukami

Atsushi Fukao

Astunori Fukuhara

Michiaki Fukui

Kenji Furukawa

Shinya Furukawa

Hiroto Furuta

Nazim Ghouri

Charlie Gilbride

Atsushi Goto

Yashdeep Gupta

Kyoung Hwa Ha

Masahide Hamaguchi

Akihiro Hamasaki

Hidetaka Hamasaki

Liyuan Han

Xiaoling Han

Toshiaki Hanafusa

Rikinari Hanayama

Masumi Hara

Goji Hasegawa

Koshi Hashimoto

Yoshitaka Hashimoto

Yuji Hataya

Akinori Hayashi

Yoshitaka Hayashi

Yasuaki Hayashino

Tatsuhito Himeno

Takumi Hirata

Takahisa Hirose

Yushi Hirota

Low‐Tone Ho

Chang Won Hong

Ruby Hoo

Mika Hori

Ichiro Horie

Yukio Horikawa

Ryota Hosomi

Tetsuya Hosooka

Cheng Hu

Chia‐Luen Huang

Chuiguo Huang

Chun‐Jui Huang

Cn Huang

Yu Huang

Chii‐Min Hwu

J Hyett

Yasuo Ido

Noriko Ihana‐Sugiyama

Katsumi Iizuka

Kaori Ikeda

Junta Imai

Kenjiro Imai

Minako Imamura

Daisuke Inoue

Tatsuhide Inoue

Junichiro Irie

Yasushi Ishigaki

Hisamitsu Ishihara

Tetsuya Ishikawa

Mohammad Safiqul Islam

Arata Itoh

Katsuomi Iwakura

Naoko Iwasaki

Yusaku Iwasaki

Masanori Iwase

Minoru Iwata

N Jahan

Zahra Jamali

T.‐S. Jap

Troels Staehelin Jensen

Jae‐Han Jeon

Xiaofan Jia

Guozhi Jiang

Sang‐Man Jin

Rozmin Jiwani

Ji Eun Jun

Elnaggar Nabil K.

Keizo Kanasaki

Akio Kanazawa

Kayo Kaneko

Shizuka Kaneko

Hideaki Kaneto

Pall Karlsson

Kenichi Kashimada

Atsunori Kashiwagi

Hideki Katagiri

Sayaka Katagiri

Naoto Katakami

Hisaya Kato

Koichi Kato

Takehiro Kato

Yoshiro Kato

Toshihide Kawai

Ryoichi Kawamura

Tomoyuki Kawamura

Daiji Kawanami

Eiji Kawasaki

Yoshiaki Kido

Takeshi Kikuchi

Touru Kikuchi

Chong Hwa Kim

Chul‐Hee Kim

Jae Hyun Kim

Nan Hee Kim

Sang Soo Kim

Sang Yong Kim

Mak Kingston

Munehiro Kitada

Tadahiro Kitamura

Atsushi Kiyota

Junji Kobayashi

Satoru Kodama

Yuzo Kodama

Mitsuhisa Komatsu

Ichiro Komiya

Tatsuya Kondo

Guilan Kong

Masahiro Koseki

Masaya Koshizaka

Daisuke Koya

Hidenori Koyama

Junji Kozawa

Naoto Kubota

Shinji Kume

Chun‐Heng Kuo

Cs Kuo

Masafumi Kurajoh

Akio Kuroda

Takeshi Kurose

Yoshiki Kusunoki

Hitoshi Kuwata

Soo‐Heon Kwak

Gormsen Lars

Seyed Mahmoud Latifi

I‐Te Lee

Jen‐Kuang Lee

Paul Lee

Seung Hwan Lee

Sung Eun Lee

Ting‐Wei Lee

Won‐Young Lee

Yongho Lee

Hong‐Yuan Li

Wen Li

Tang Yi Liao

Lee‐Wai Lim

Soo Lim

Che Lin

Chia‐Hung Lin

Chia‐Huei Lin

Kun‐Der Lin

Liang‐Yu Lin

Shin‐Yu Lin

Yen‐Chung Lin

Jian‐Ping Liu

Liping Lu

Peirong Lu

Shao‐Gang Ma

Norikazu Maeda

Shiro Maeda

Akhil Maheshwari

Noboru Makabe

Rayaz Malik

Oreste Marrone

Takayuki Masaki

Dasaku Masuda

Munehide Matsuhisa

Takeshi Matsumura

Takashi Matsuoka

Shu Meguro

Otsuki Michio

Takashi Miida

Takashi Miki

Takayuki Miki

Maria Mirabelli

Parvin Mirmiran

Tomoya Mita

Junnosuke Miura

Teruki Miyake

Takeshi Miyatsuka

Ayako Miyazaki

Hideaki Miyoshi

Hiroki Mizukami

Masami Mizuno

Mie Mochizuki

Ricardo Mora‐Rodriguez

Jun Mori

Yasumichi Mori

Yusaku Mori

Katsutaro Morino

Tomoaki Morioka

Yoshiaki Morishita

Tatsumi Moriya

Imane Motaib

Shota Moyama

Seiichi Munesue

Chieko Murakami

Takaaki Murakami

Koji Murao

Takashi Murata

Seiho Nagafuchi

Yoshio Nagai

Mototsugu Nagao

Shoichiro Nagasaka

Kei Nakajima

Akinobu Nakamura

Shuhei Nakanishi

Yujiro Nakano

Eitaro Nakashima

Ming‐Yen Ng

Qin Xiang Ng

Roy Ng

Masahiro Nishi

Yuichiro Nishida

Rimei Nishimura

Hitoshi Nishizawa

Kousuke Noda

Mitsuhiko Noda

Hiroyuki Nodera

Takashi Nomiyama

Masatoshi Nomura

Shinsuke Noso

Yoshihiro Ogawa

Masahito Ogura

Mica Ohara‐Imaizumi

Toshiaki Ohkuma

Mitsuru Ohsugi

Yasuharu Ohta

Yoichi Oikawa

Yosuke Okada

Tsuyoshi Okura

Yukiko Onishi

Hiroshi Onuma

Vahidreza Ostadmohammadi

Nobuaki Ozaki

Maria Ruth B Pineda‐Cortel

Paola Pontrelli

Xingwu Ran

Leanna Ross

Hamza Saad

Atsuhito Saiki

Yoshifumi Saisho

Kazuhiko Sakaguchi

Hiroshi Sakaue

Kenichi Sakurai

Kazunori Sango

Motoaki Sano

Daisuke Sato

Eisuke Sato

Junko Sato

Hiroaki Satoh

Takafumi Senokuchi

Yunfeng Shen

Kenichi Shikata

Michio Shimabukuro

Akira Shimada

Hitoshi Shimano

Dai Shimono

Ichiro Shiojima

Jun Shirakawa

Takuhito Shoji

Khalid Siddiqui

Masakatsu Sone

Noriyuki Sonoda

Vedantham Srinivasan

Julie Stoy

Kazuhiro Sugimoto

Ken Sugimoto

Taiki Sugimoto

Yoshihisa Sugimura

Takehiro Sugiyama

Atushi Suzuki

Ryo Suzuki

Shigeru Suzuki

Yuji Tajiri

Mitsuyoshi Takahara

Hirokazu Takahashi

Kazuma Takahashi

Iseki Takamoto

Takeshi Takayanagi

Minoru Takemoto

Yumi Takiyama

Yoshikazu Tamori

Yoshifumi Tamura

Elise Chia‐Hui Tan

Katsuya Tanabe

Daisuke Tanaka

Nagaaki Tanaka

Nobue Tanaka

Toru Tanigaki

Hirotaka Tashiro

Yasuo Terauchi

Kj Tien

Kazuyuki Tobe

Atsuhito Tone

Keiichi Torimoto

Masao Toyoda

D Tsilingiris

Kyoichiro Tsuchiya

Satoru Tsujii

Shin Tsunekawa

Yuya Tsurutani

Yasuko Uchigata

Satoshi Ugi

Perez‐Zepeda Mario Ulises

Hiroyuki Unoki‐Kubota

Emi Ushigome

Isao Usui

Ryota Usui

Shinichi Usui

Prashanth Vas

Mislav Vrsalovic

Jun Wada

Chaofan Wang

Chih Yuan Wang

Jun‐Sing Wang

Shu‐Huei Wang

Xiao Qun Wang

Zilian Wang

Jong Chul Won

Chung‐Ze Wu

Zekai Wu

Wang‐Hong Xu

Hiroaki Yagyu

Eijiro Yamada

Sho‐Ichi Yamagishi

Takeshi Yamamoto

Yasuhiko Yamamoto

Yukiyo Yamamoto

Shunsuke Yamane

Tomoya Yamashita

Mika Yamauchi

Yuji Yamazaki

Jinhua Yan

Yuan‐Horng Yan

Toshihiko Yanase

Jichun Yang

Xuefeng Yang

Hisafumi Yasuda

Ichoro Yasuhi

Hiroshi Yatsuya

Hyon‐Seung Yi

Norihide Yokoi

Hiroshi Yokomichi

Masayasu Yoneda

Takashi Yoneda

Hideto Yonekura

Tohru Yorifuji

Yu Yuan

Fujita Yukihiro

Bedowra Zabeen

Sueziani Zainudin

Seyed Mohsen Aghaei Zarch

Sen Zhang

Xiaomin Zhang

Yan Zhang

Zhongheng Zhang

Xihai Zhao

Haoming Zhou

Wensheng Zhou

Yimin Zhu

Tali Zitman‐Gal

